# A novel scoring system to guide prognosis in patients with pathological fractures

**DOI:** 10.1186/s13018-018-0931-x

**Published:** 2018-09-06

**Authors:** Xiang Salim, Peter D’Alessandro, James Little, Kulvir Mudhar, Kevin Murray, Richard Carey Smith, Piers Yates

**Affiliations:** 1 Department of Orthopaedics, Sir Charles Gairdner Hospital, 55 Viewway Nedlands, Perth, WA 6009 Australia; 2 Department of Orthopaedics, Fiona Stanley Fremantle Hospital Groups, Perth, Australia; 3 Department of Orthopaedics, Royal Perth Hospital, Perth, Australia; 4 Centre for Applied Statistics, University of Western Australia, Perth, Australia; 5 Orthopaedic Research Foundation of Western Australia (ORFWA), Perth, Australia

**Keywords:** Pathological fracture, Metastases, Scoring system, Survival probability

## Abstract

**Background:**

The most appropriate treatment of pathological fractures from metastatic disease depends on several factors, one of the most important being predicted life expectancy. The aim of this study was to identify the variables that influence prognosis and utilise these to develop a novel scoring system to better predict life expectancy post-pathological fracture.

**Methods:**

The records of all patients that presented with metastatic pathological fractures over a 10-year period from the only tertiary orthopaedic departments in Western Australia were retrospectively examined. Variables assessed were primary cancer type, fracture site, fixation method, cement augmentation, pre-morbid level of physical functioning, complication rate, treatment with chemotherapy or radiotherapy and appendicular, spinal and visceral metastatic load.

**Results:**

A total of 233 patients were included. Median survival from fracture to death was 4.1 months. Median time from cancer diagnosis to pathological fracture was 14.2 months. There was a statistically significant association between patient survival and primary cancer type, physical functional score, spinal metastatic burden and use of chemotherapy or radiotherapy.

**Conclusion:**

A novel scoring system has been developed that offers a survival probability based on patient’s individual circumstances. This can guide specialist management and offer patients a more accurate expectation of functional outcome and survival time.

## Background

As the average life expectancy has increased, so too has the prevalence of cancer [1, 2]. Recent advances in diagnostic and therapeutic capabilities have resulted in a better prognosis in many cancer patients [3]. Approximately 10% of patients with bony metastases will suffer a pathological fracture at some point during their clinical course [1, 4, 5]. Pathological fractures have significant implications for patient morbidity and mortality and are often considered a marker of end-stage cancer. The literature suggests a 1-year survival rate in the range of 30–40% [6–9].

A number of studies have identified significant variables in patients with metastatic lesions and how they relate to patient prognosis [4, 8–12]. However, this study differs by identifying the significant variables at time of pathological fracture, which is often the point at which the surgical team are first involved.

When considering surgical management of a pathological fracture, the key operative goals include pain relief, early mobilisation and minimal morbidity and complications [2, 5, 13]. The chosen implant and construct should be able to withstand the patient’s expected level of activity and appropriately match their expected survival [2, 5].

Although important, the documented ability of clinicians to predict prognosis in patients with metastatic bone disease is poor, with reported accuracy of only 18% reported in the literature [11]. In response to this, we sought to develop a novel scoring system based on the statistically significant variables identified at the time of pathological fracture that can be utilised to more accurately predict prognosis and overall survival. Such a scoring system has never before been developed in patients who suffer a pathological fracture, and has the potential to guide surgical management and provide a more evidence-based patient expectation.

## Methods

All records from patients admitted with a pathological fracture over a 10-year period (2002–2012) to Fremantle, Sir Charles Gairdner and Royal Perth Hospital in Western Australia were retrospectively analysed. Inclusion criteria were pathological fractures secondary to metastatic bone disease. Exclusion criteria were primary bone tumours, spinal pathological fractures, paediatric patients (< 18 years) and peri-prosthetic fractures. Two hundred thirty-three patients that met these criteria were identified.

Recorded variables included age, sex, primary cancer, fracture site, method of fixation, use of cement augmentation, appendicular metastatic load, spinal metastatic load, presence of visceral metastases, co-morbidities, functional scoring before and after the fracture has occurred (Eastern Cooperative Oncology Group (ECOG) score) [14], post-operative complications and use of chemotherapy and radiotherapy. ECOG score was obtained by review of allied health notes and recording pre-injury and best post-operative functional score. The metastatic load was measured through review of existing imaging including plain film, computed tomography (CT), magnetic resonance imaging (MRI), bone scan and positron emission tomography (PET). Metastatic lesions were counted and recorded as 0, 1, 2 or 3 or more to axial and appendicular skeleton as well as viscera.

Statistical analysis of time to death from fracture and the time between cancer diagnosis and fracture was carried out using Cox proportional hazards modelling. Multivariate hazard ratios (HRs) and 95% confidence intervals (CIs) are presented for only those variables that were retained and statistically significant in the final model. Change in ECOG scores (pre- to post-operatively) was analysed using multiple linear regression. In all models, model selection was carried out retaining significant predictors in the final model using a 0.05 significance level. The Cox proportional hazards regression model was used to construct a nomogram, providing a visual representation of our scoring system. All data was analysed using the R environment for statistical computing [15].

## Results

Analysis of the combined hospital database identified 233 patients from Sir Charles Gairdner Hospital (*n* = 89), Fremantle Hospital (*n* = 72) and Royal Perth Hospital (*n* = 72). Table 1 provides an outline of the basic demographics and a breakdown of several key variables investigated in this group. Primary cancer type was predominantly breast and lung (29% and 21% respectively). The majority of fractures were at the proximal femur and humerus (56% and 25% respectively).

**Table 1:** Demographics and distribution of study group

Variable	Category	Number (%)
Age	< 60	53 (23)
	60–74	94 (40)
	75+	86 (37)
Gender	Female	124 (53)
	Male	109 (47)
Primary cancer	Breast	69 (29)
	Lung	49 (21)
	Other	56 (24)
	Prostate	32 (14)
	Renal	27 (12)
Spinal metastases	0	70 (30)
	1, 2, 3	163 (70)
Appendicular metastases	0, 1, 2	106 (45)
	3	127 (55)
Visceral metastases	Missing	11 (5)
	No	71 (30)
	Yes	151 (65)
Fracture site	Humerus	58 (25)
	Proximal femur	131 (56)
	Distal femur	24 (10)
	Other	20 (9)
Treatment	Non-operative	25 (11)
	Plate fixation	27 (12)
	Intramedullary nail	114 (49)
	Arthroplasty	67 (29)
Chemotherapy	Missing	14 (6)
	No	75 (32)
	Yes	144 (62)
Radiotherapy	Missing	13 (6)
	No	64 (27)
	Yes	156 (67)

### Diagnosis to fracture

The median time from cancer diagnosis to pathological fracture was 14.2 months (IQR 1.8–57.3). For 40 patients (17.1%), the pathological fracture itself was the presenting event for a malignant diagnosis. When examining the time from diagnosis to fracture, primary cancer type was a statistically significant variable with lung cancer having the worst prognosis and breast cancer the best (HR for lung to breast = 5.70, 95% CI 3.66–8.88). After adjusting for cancer type distribution, gender was also statistically significant with males having a higher event risk compared to females (HR 1.69, 95% CI 1.28–2.25). Treatment with chemotherapy was statistically significant in delaying time from cancer diagnosis to pathological fracture (HR, 1.46 95% CI 1.09–1.95) while radiotherapy was not. The overall median follow-up time from cancer diagnosis to death was 26.6 months (IQR 6.7–72.8).

### Fracture to death

The median time from fracture to death in all comers was 4.1 months (IQR 1.6–12.7). When examining the time to death from fracture, the variables primary cancer type (*P* < 0.001), ECOG pre-fracture score (*P* = 0.004), chemotherapy (*P* = 0.003), radiotherapy (*P* < 0.001) and spinal bone metastases (*P* = 0.004) were all statistically significant in the final multivariate model.

**Fig. 1:**
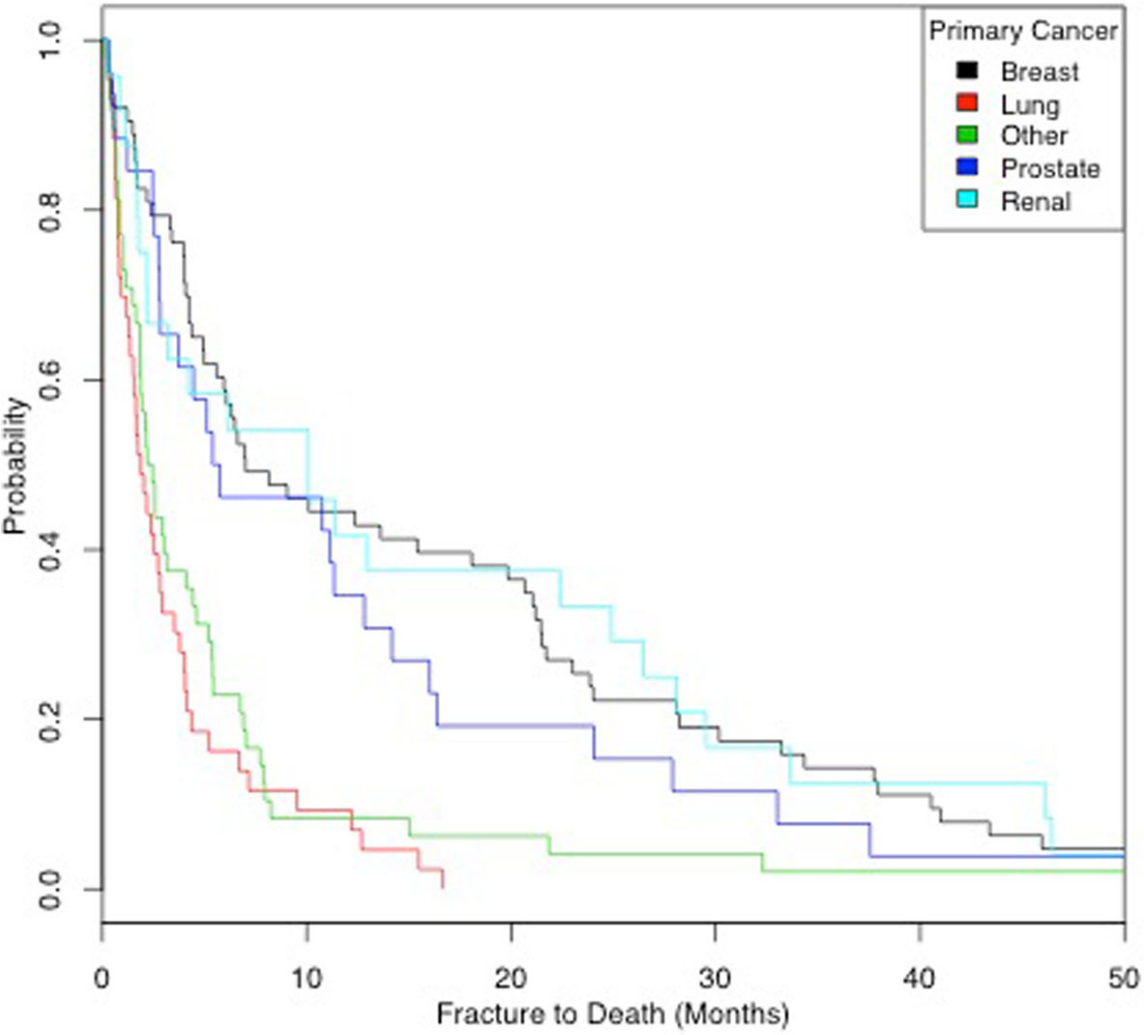
Kaplan-Meier analysis of the survival rates of each primary cancer type

Breast cancer had the best survival outcome and lung cancer the worst (HR lung to breast = 4.29, 95% CI 2.74–6.71) (*P* < 0.001). Between fracture and death, the median survival duration for breast cancer was 7 months (IQR 4–24), which compares well against lung cancer with a median survival 1.87 months (IQR 0.8–4). Figure 1 shows a Kaplan-Meier analysis of the survival rates of each primary cancer. All patients with lung cancer were deceased 17 months after fracture.

### ECOG

Pre-fracture ECOG score was statistically significant when analysing time from fracture to death (*P* = 0.004). An ECOG score of 0 suggests that a disease process does not alter physical functioning whilst a score of 4 implies that the patient is completely disabled and bound to bed or chair [14]. Patients with a higher pre-fracture ECOG had a poorer prognosis when compared to those with a score of 0 (HR 1.58, CI 95% 1.16–2.14).

**Fig. 2:**
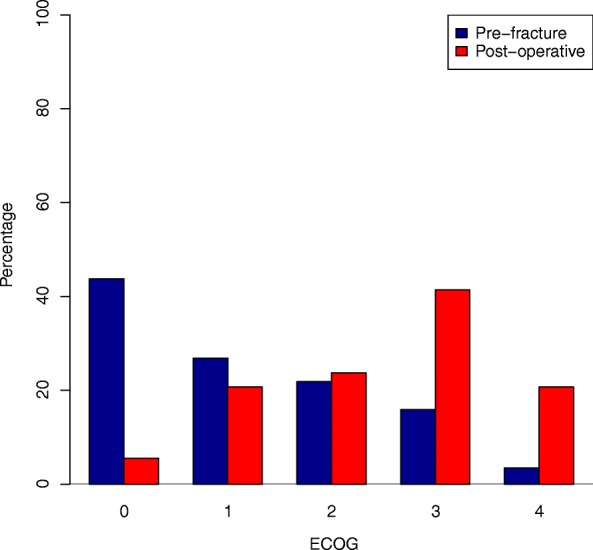
Distribution of ECOG score pre-fracture and post-operation

Figure 2 shows the distribution of ECOG score pre-fracture and post-operation. As expected, the majority of subjects had an increase in ECOG score (75.7%), indicating a worse post-operative level of functioning. Noticeably, nine cases (4.1%) moved from an ECOG score of 0 to a score of 4. A statistical analysis on change in ECOG score (while adjusting for pre-treatment score) found that the statistically significant predictors of a worsening in ECOG score included fracture site (*P* = 0.008) and treatment with chemotherapy (*P* = 0.009), with those receiving chemotherapy having a smaller change in ECOG. When examining fracture site, those with a humeral fracture had a smaller loss of function than those with a fracture at proximal or distal femur (*P* = 0.001 and *P* = 0.017 respectively).

### Spinal, appendicular and visceral metastases

There were no spinal metastases in 30% of cases; however, 53.7% of cases had three or more. The number of spinal metastases was shown to be statistically significant in predicting time from fracture to death (*P* = 0.004). Those who had any spinal metastases had a shorter survival time when compared to those who had none (HR 1.65, 95% CI 1.18–2.31).

A large proportion (54.5%) of cases had three additional appendicular bone metastases, while 14.2% had zero. There was no statistically significant relationship between appendicular or visceral metastatic load and survival rate post-fracture.

### Fracture site, method of fixation and use of cement augment

Fracture site was predominantly proximal femur and humerus (56% and 25% respectively). Treatment included intramedullary nail, plate fixation and arthroplasty (49%, 12% and 29% respectively) with 10% of patients were managed non-operatively. Fracture site, method of fixation and use of cement augmentation did not have a statistically significant impact on survival post-fracture.

### Chemotherapy and radiotherapy

Chemotherapy and radiotherapy treatment was present in 61.8% and 66.95% of patients. Patients who did not receive chemotherapy had a higher risk of mortality than those who did (HR 1.74, 95% CI 1.21–2.49) (*P* = 0.003). Similarly, radiotherapy was also protective in survival post-fracture (HR 1.79, 95% CI 1.27–2.51) (*P* < 0.001). Median extended survival duration with use of chemotherapy and radiotherapy was 3.56 and 3.78 months respectively.

### Survival probability score

**Fig. 3:**
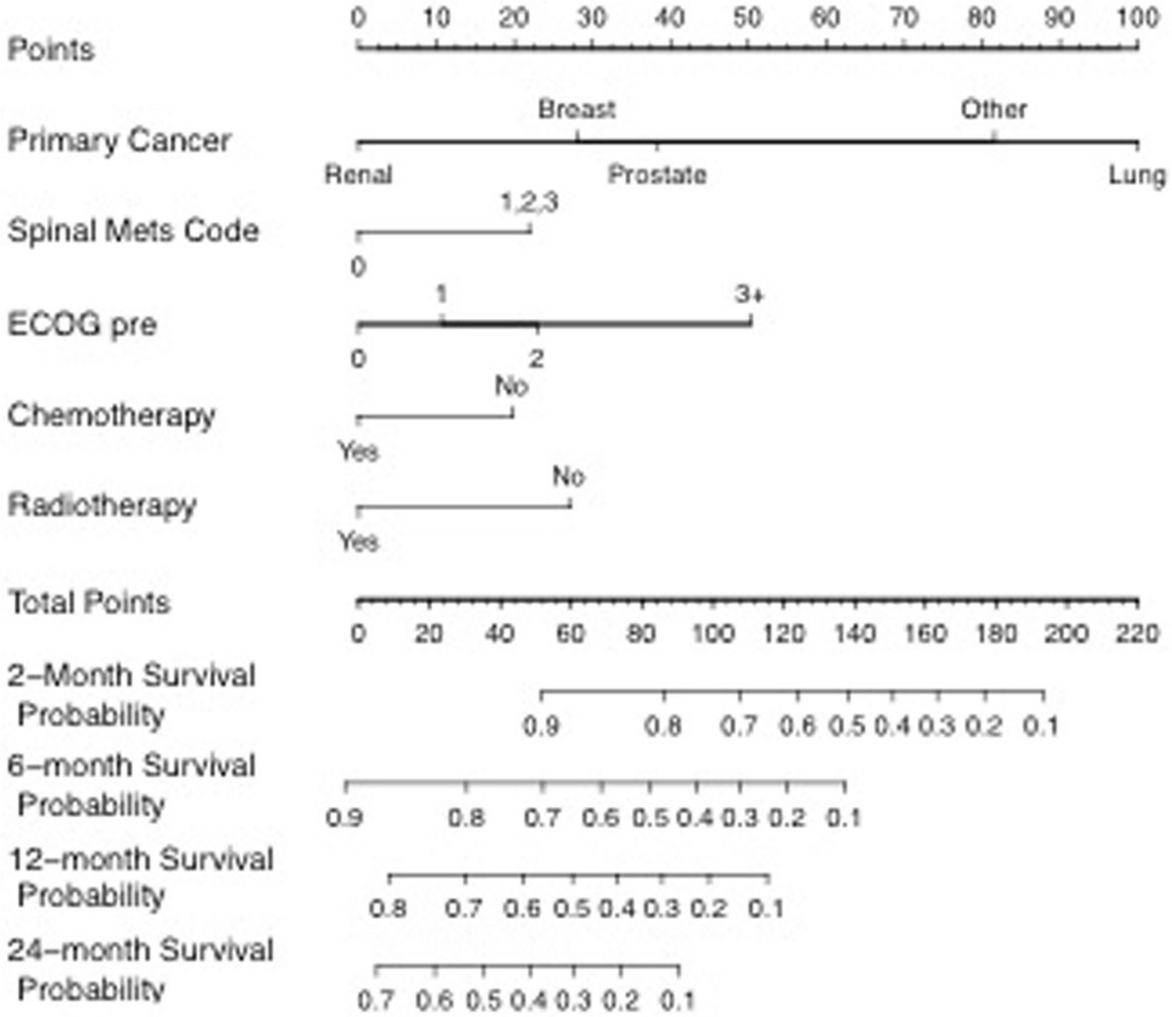
Nomogram representation of our scoring system which provides different survival probabilities for a range of survival times based on individual patient characteristics

With our large sample size drawn across an entire population base along with the incorporation of statistically significant variables, our team was able to develop a novel and unique scoring system for these patients. This has never before been available for patients at time of pathological fracture and can be used to provide a more accurate assessment of prognosis and survival. This is depicted in Fig. 3 where a nomogram gives a visual representation of our scoring system which provides different survival probabilities for a range of survival times based on individual patient characteristics. This is yet to be validated as our team elected to use all 233 available cases to generate a sufficiently powered scoring system, rather than divide the group into a subset for generating and a subset for validating.

**Fig. 4:**
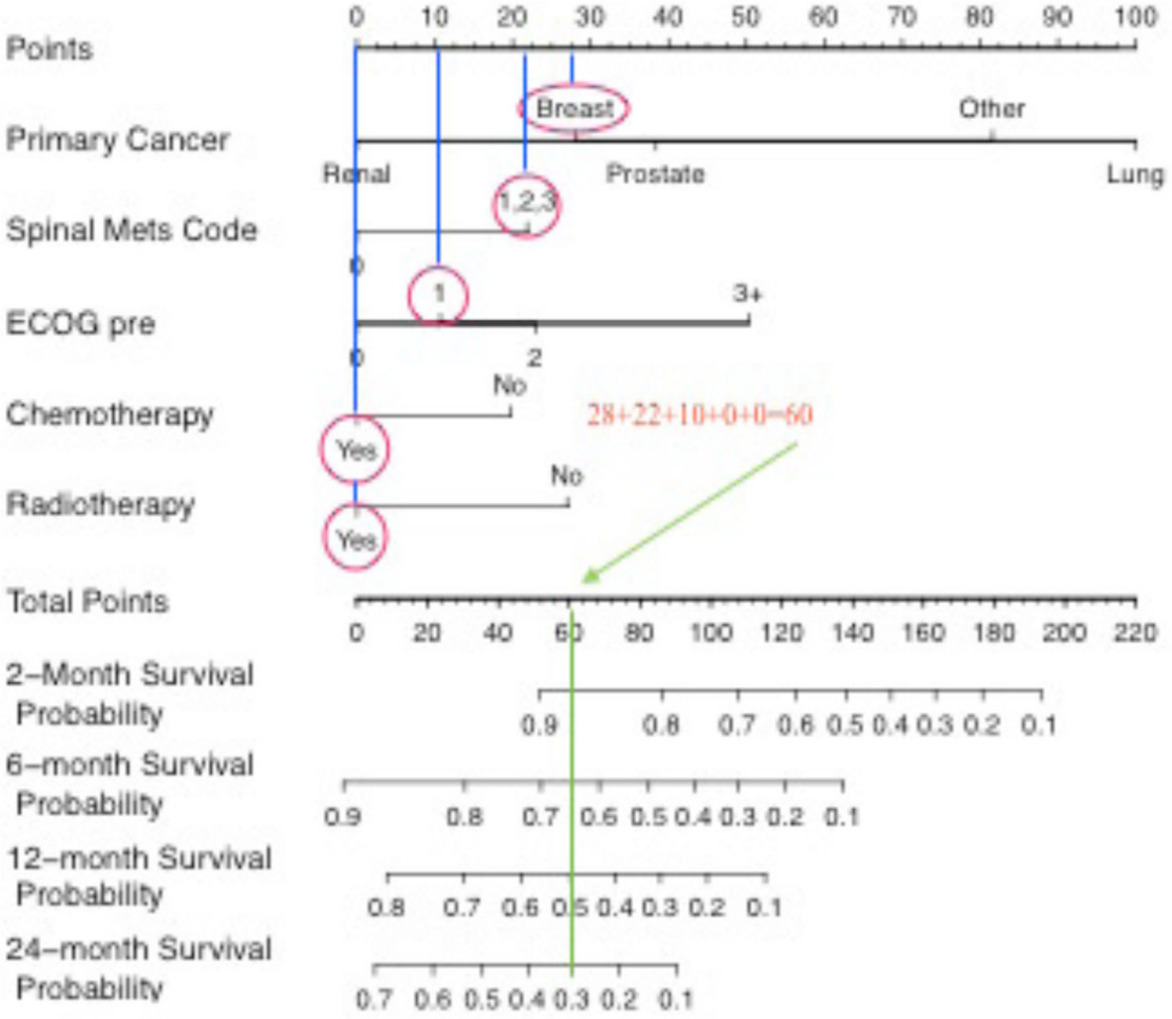
Example case of a pathological fracture of breast cancer origin, with spinal metastases, a pre-injury ECOG of 1, who has had chemotherapy and radiotherapy treatment. Circle relevant variable (red). Draw line up to attribute point (blue). Add each variable point to find the sum (orange). Find total point value and draw line down to identify the survival probability at each time (green)

To use the nomogram, each variable is identified on the left and the individual patient’s characteristic circled. A vertical line is then drawn up to intersect the ‘points’ axis to determine the point score for each variable. The total sum of points is then calculated. This total sum value is located on the ‘total points’ axis, and a vertical line is drawn down. Survival probability at each time period (2/6/12/24 months) is determined by the point at which this line intersects it. As an example, Fig. 4 shows the process for a patient with a pathological fracture of breast cancer origin, with spinal metastases, a pre-injury ECOG of 1, who has had chemotherapy and radiotherapy treatment.

## Discussion

During the natural course of the disease process, malignancies commonly metastasise to the bone [1, 4]. Many studies have examined the variables that influence progression of metastatic lesions to pathological fracture [1, 4, 5, 7]; however, few studies have investigated the factors that impact morbidity and mortality once the pathological fracture has occurred.

This study is a state-wide expansion on the pilot paper published by our institution [13]. Our preceding pilot article was the first to document survival time following pathological fracture and was able to identify variables that significantly influenced prognosis [13]. However, until now, there has been no conclusive scoring system available to help predict prognosis at time of pathological fracture. This is an important and novel development, as better insight into prognosis would help treating clinicians identify patients with poorer or favourable survival prospects and therefore guide surgical treatment options.

Our study found that the primary cancer type had a statistically significant impact on time from cancer diagnosis to pathological fracture. Demographic primary cancer type was mainly of breast and lung origin which is consistent with existing literature [6, 10, 11]. Breast and prostate had the longest time to pathological fracture with a median delay of 49.2 (IQR 11.7–115.6) and 29 months (IQR 12.2–59.4) respectively. In contrast, lung cancer had the shortest time to fracture with a median of just 2.1 months (IQR 0–6.8). Given the shorter period from diagnosis to fracture, we recommend more frequent screening in this subgroup so as to detect significant metastatic bone lesions before they fracture. This is especially important given the poor prognosis following a fracture in this subgroup. After adjusting for primary cancer type distribution, gender was also statistically significant with males having a higher event risk compared to females in time from diagnosis to fracture. This is difficult to interpret, but may be related to males typically seeking medical attention later in a disease course which may result in a delay to cancer diagnosis.

Median survival post-fracture in our expanded cohort was 4.1 months with a 1-year survival rate across all patients of 27%. While slightly better than the survival described in our pilot paper (3.3 months), this is still a group of patients with an incredibly poor overall prognosis that has perhaps been underestimated in previous literature [6–9]. Lung cancer median survival was much worse at 1.87 months when compared to breast (7 months), prostate (5.24 months) and renal (10.09 months) median survival. This variability of prognosis in different primary cancers is well recognised in existing literature [1, 5, 8, 10, 11, 16, 17]; however, it has never been incorporated into a scoring system to predict patient prognosis post-pathological fracture as it has in this paper.

Negative prognostic factors in patients with metastatic bone disease include primary lung cancer, metastatic load, visceral metastases, pathological fracture and poor functional performance score [8, 10, 11]. Katagiri et al. developed a scoring system for patients with bony metastases [18]. This differed from our team’s goal of identifying prognostic factors post-pathological fracture. Their team found that primary cancer site, ECOG score, presence of visceral and cerebral metastases, any previous chemotherapy and multiple skeletal metastases were significant prognostic factors. Interestingly, their team found chemotherapy to be a negative prognostic factor, which likely represents a treatment selection bias. Furthermore, their findings suggested that pathological fractures are not a negative prognostic factor in patients with metastatic bone disease, which differs from what most evidence suggests [8, 10, 11].

An article by Nathan et al. also examined the biochemical factors surrounding prognosis and found that low pre-operative haemoglobin, albumin and white cell count were all independent negative prognostic factors [11]. This paper emphasised the difficulty in estimating mortality rate in patients with metastatic bone disease, as only 18% of clinician estimates were accurate in predicting actual survival [11].

Multiple scoring systems exist for patients with spinal metastases, which attempt to predict post-operative prognosis so as to rationalise management decisions [10, 12, 19]. The sentinel paper by Tokuhashi et al. that first developed a scoring system in patients with spinal metastases was the initial inspiration for our study into appendicular pathological fracture prognosis and guided the choice of variables we would investigate [12]. A more recent paper by Dardic et al. evaluated these scoring systems and corroborated that visceral metastases, primary tumour type, functional performance score and number of spinal metastases all significantly influenced survival [20]. The prognostic relevance of spinal metastatic burden in patients with an appendicular pathological fracture has not been previously studied.

Tsuda et al. recently conducted an investigation assessing the factors affecting post-operative complications and short-term mortality after surgery specific to femoral pathological fractures [2]. They found that post-operative complications were significantly associated with older age, primary tumour type, higher Charlson Comorbidity Index and blood transfusion [2]. In addition, they concluded that 30-day mortality was significantly higher in patients with rapid-rapid growth tumours, visceral metastases, internal fixation method and no post-operative chemotherapy [2].

Our study found that primary cancer type, pre-fracture ECOG score, spinal metastatic burden and treatment with chemotherapy and radiotherapy were statistically significant variables in the survival rate post-pathological fracture. Age, complications, gender, fixation method, fracture site and visceral and appendicular metastases were not found to be significant factors.

Spinal metastases had a statistically significant effect on patient outcome following pathological fracture. Our pilot study was the first to describe the spinal metastatic burden as a prognostic variable in patients with appendicular bony metastases. Our expanded study has also found it to be a significant predictive variable and one that should be considered in patients with appendicular pathological fractures.

Visceral and appendicular metastatic load was not a statistically significant prognostic factor in our study. This is in contrast to existing evidence in patients with metastatic bone disease that suggest a greater appendicular or visceral metastatic burden to be a negative prognostic factor [1, 4, 8–11]. We believe this finding reflects the different points on the pathological spectrum, where pathological fractures are further progressed and are often considered end-stage markers. Our results suggest that at point of pathological fracture, as the disease process is so advanced, the number of visceral or appendicular metastasis are not relevant to survival prognosis.

The use of chemotherapy and radiotherapy was found to have a significantly positive effect on survival rate following a pathological fracture. Interestingly, it was observed that use of chemotherapy also prolonged the time between diagnosis and pathological fracture, whereas use of radiotherapy did not. This likely reflects the systemic nature of chemotherapy as oppose to the targeted local effects of radiotherapy. It was also observed that patients who received chemotherapy had a significantly smaller change in ECOG post-operatively. These findings corroborate existing evidence advocating use of adjuvant chemotherapy and radiotherapy. In Australia, these adjuvant therapies are commonly available; however, not all patients receive adjuvant treatment. In our study, a surprising 32.19% and 27.47% did not receive chemotherapy and radiotherapy respectively. Furthermore, in certain developing countries, these treatments are less available due to financial and logistical reasons. For these reasons, use of adjuvant therapy is included in our prognosticating scoring system.

Pre-fracture ECOG score statistically significantly influenced prognosis as patients with a better functional score prior to pathological fracture lived longer following surgery. These findings are consistent with existing literature supporting the impact of pre-morbid physical function on post-operative function and survival time [1, 8, 9, 11, 16]. This is intuitive; however, it emphasises the importance of optimising and maintaining cancer patients’ functional mobility and independence at both the pre-injury and post-operative stage.

Our study’s limitations reflect the nature of the retrospective audit design. Our collection of ECOG score was dependent on the assessment and documentation of several different occupational therapists. Similarly, the operation itself was performed by different surgeons of differing levels of experience across three hospital sites. Finally, the heterogeneity of our cohort and their primary tumour type means a wide variety of chemotherapy and radiotherapy regimes prescribed of which the binary yes/no analysis likely oversimplifies. Despite these limitations, we have found a statistically significant relationship between prognosis following pathological fracture and several variables including primary cancer type, pre-fracture ECOG score, spinal metastatic burden and use of chemotherapy and radiotherapy. Using these significant variables, we have developed a novel scoring system which can be used to estimate survival probability at time of pathological fracture.

## Conclusion

This expanded study has included a large cohort of patients, with more than triple the number of patients described in the original pilot paper within the same 10-year time frame. It is adequately powered to find statistical significance in several of our key variables of interest. Due to the relative geographical isolation of Western Australia and the inclusion of every tertiary referral centre in the state, it is likely that the vast majority of patients presenting with pathological fractures in this community have been included.

We have been able to develop a novel scoring system that can be utilised to estimate survival probability based on these statistically significant variables. This will enable treating clinicians to more accurately estimate survival time, which is often a source of great anxiety to both the patient and their family. An important principle in the management of patients with a pathological fracture is that the patient should live longer than the time needed to recover and rehabilitate from the operation. A more evidence-based estimate of prognosis will be an invaluable tool in guiding the treating team in management decisions. This paper will fill a significant void in the literature; as to our knowledge, there is no existing scoring system of this nature currently available.
